# Response to letter to the editor regarding the article “Complementary role of computed tomography texture analysis for differentiation of pancreatic ductal adenocarcinoma from pancreatic neuroendocrine tumors in the portal-venous enhancement phase”

**DOI:** 10.1007/s00261-020-02816-9

**Published:** 2020-10-17

**Authors:** Christian Philipp Reinert, Marius Horger

**Affiliations:** 1 Department of Diagnostic and Interventional Radiology, University Hospital Tübingen, Hoppe-Seyler-Str.3, 72076 Tübingen, Germany; 2 Department of Diagnostic and Interventional Radiology, Eberhard-Karls-University, Hoppe-Seyler-Str.3, 72076 Tuebingen, Germany

We are honored to learn that our report [1] has been positively received by the colleagues Dr. Shuai Ren and Dr. Zhongqui Wang.

Indeed, in 4/42 patients with pancreatic neuroendocrine neoplasms (PNEN), the tumors were classified as hyperdense in the portal-venous enhancement phase. Following your recommendation, we performed an additional analysis for differentiation between pancreatic ductal adenocarcinomas (PDAC) and PNEN under exclusion of these 4 patients. Regarding first-order textural features, statistical analysis revealed a significantly lower “10th percentile” and “90th percentile” in PDAC compared to PNEN (*p* < 0.01), and significantly higher “total energy” and “minimum” in PNEN (*p* < 0.001). The second-order Gray-Level co-occurrence Matrix feature “Informational Measure of Correlation (Imc2)” proved significantly higher in PDAC compared to PNEN (*p* < 0.001). The first-order features “median,” “maximum” and “energy” showed a tendency toward lower values in PDAC compared to PNEN, now without statistical significance (*p* > 0.05).

Regarding the role of CTTA in differentiating G1 from G2/3 tumors and not—as recommended by Dr. Ren and Dr. Wang—in differentiating G1/2 from G3 tumors, we aimed to address this issue staying conform to a previous, similar work [2] in order to demonstrate that our results are indeed comparable with those of Choi et al. and therefore evidently applicable in this clinical setting. From a clinical standpoint, we understand and accept this criticism. However, this topic was new and we thought that making these results more comparable with those of Choi et al. would increase the acceptance for the use of this technique.
